# Autonomous Sea Floor Coverage with Constrained Input Autonomous Underwater Vehicles: Integrated Path Planning and Control

**DOI:** 10.3390/s25041023

**Published:** 2025-02-09

**Authors:** Athanasios K. Gkesoulis, Panagiotis Georgakis, George C. Karras, Charalampos P. Bechlioulis

**Affiliations:** 1 Department of Electrical and Computer Engineering, University of Patras, 26504 Patras, Greece; gkesoulisth@upatras.gr (A.K.G.); up1067233@ac.upatras.gr (P.G.); chmpechl@upatras.gr (C.P.B.); 2 Athena Research Center, Robotics Institute, 15125 Marousi, Greece; 3 Department of Informatics and Telecommunications, University of Thessaly, 35100 Lamia, Greece

**Keywords:** coverage path planning, autonomous underwater vehicles, robust control, input saturation, ROS, Gazebo, BlueROV2, underwater robotics

## Abstract

Autonomous underwater vehicles (AUVs) tasked with seafloor coverage require a robust integration of path planning and control strategies to operate in adverse real-world environments including obstacles, disturbances, and physical constraints. In this work, we present a fully integrated framework that combines an optimal coverage path planning approach with a robust constrained control algorithm. The path planner leverages a priori information of the target area to achieve maximal coverage, minimize path turns, and ensure obstacle avoidance. On the control side, we employ a reference modification technique that guarantees prescribed waypoint tracking performance under input constraints. The resulting integrated solution is validated in a high-fidelity simulation environment employing ROS, Gazebo, and ArduSub Software-in-the-Loop (SITL) on a BlueROV2 platform. The simulation results demonstrate the synergy between path planning and control, illustrating the framework’s effectiveness and readiness for practical seafloor operations such as underwater debris detection.

## 1. Introduction

Marine pollution, mainly composed of synthetic polymers [1,2], the need to regularly inspect vital underwater infrastructures, such as pipelines and cables [3], and the exploration of the deep sea, which represents the least explored area of Earth [4], underscore the importance of accurate seafloor coverage and mapping. Traditional methods reliant on divers or remotely operated vehicles are costly, time-consuming, and potentially hazardous. In contrast, autonomous underwater vehicles can offer a more efficient and secure solution [5]. However, the ability to operate independently and continuously in remote and adverse environments is still an open topic and poses great technical challenges.

### 1.1. Optimal Coverage Path Planning

To this effort, an issue of central importance is the development of robust path planning and control strategies that enable AUVs to navigate complex terrains, completely cover regions of interest avoiding obstacles, and counter disturbances such as undersea currents. Coverage Path Planning (CPP) is the problem of determining a trajectory that ensures full coverage of a target area while avoiding obstacles. It is a fundamental task in various undersea and oversea applications such as underwater exploration [6], mosaicked imaging of the ocean floor [7], inspection of complex structures [8], autonomous cleaning [9], agricultural operations [10], and search and rescue missions [11]. The main requirements for a CPP algorithm are ensuring a complete coverage of the target area, avoiding overlapping of paths and collision with obstacles, providing as simple as possible trajectories, and applying optimality criteria when possible [12]. A variety of CPP strategies have been developed to address the requirements of robotic applications. CPP methods are categorized into heuristic, complete, offline, and online approaches [13]. Heuristic methods and complete algorithms differ on whether they are provable or not. Offline approaches assume prior knowledge of the environment, whereas online methods leverage real-time sensor data to adjust paths dynamically.

A prevalent approach to CPP is the exact cellular decomposition methodology. It consists of a method to decompose the target area into cells, a second method used to cover each cell, and a third one that computes a sequence to visit each cell exactly once. The two main decomposition strategies are trapezoidal and boustrophedon [12]. The trapezoidal decomposition is an offline method that decomposes the target area into trapezoidal cells. The coverage of each trapezoidal cell can be achieved through simple back-and-forth motions and the complete coverage is ensured by guaranteeing an exhaustive walk through all cells. However, the path length provided by trapezoidal decomposition becomes longer with the number of cells, which is the main disadvantage of such methods. In seminal work [14], boustrophedon decomposition was introduced to overcome this limitation of the trapezoidal decomposition. The main advantage is that it reduces the number of cells and therefore the length of the obtained paths. Other approaches include Morse methods, Grid- and Graph-based coverage, and Reinforcement Learning, and the interested reader is referred to the survey papers [12,15,16].

Another ubiquitous topic in the CPP literature is ensuring optimality of the constructed paths. A measure of optimality is the length and/or the completion time of the path and is possible only for target areas that are a priori fully or partially known [12]. For 2D spaces, a well-known optimal line-sweep based method was presented in [17]. The central optimality idea is to minimize the number of turns of the path as each turn is associated with added energy cost for the robot. This is achieved by choosing an appropriate sweep direction for each cell that corresponds to the normal of the minimum generalized width direction of the cell. The algorithm is then completed by utilizing dynamic programming to minimize the cost of visiting every cell. The idea of optimality through minimizing the number of turns is further investigated in work [18], where the turns are further categorized and associated with different costs. In work [19], the authors generalize the idea of [17] to non-convex polygons and follow a global approach to the minimization. A method for calculating the minimum generalized width for non-convex cells is developed that is iterated to become more optimized and parallel line segments are placed in each region. Then, a minimum-length tour of the segments is computed via posing the problem as a generalized trading salesman one and using Dubins’ car model as a means to calculate transition costs between segments. A brief comparison of the line-sweep based CPP methodologies is provided in Table 1.

### 1.2. Prescribed Performance Control with Input Constraints

Building on the foundations of CPP, the integration of robust control strategies is pivotal to addressing the challenges of autonomous coverage in real-world robotic applications. Autonomous robots often operate in highly dynamic and uncertain environments, and robust control algorithms can ensure the system’s ability to adhere to the planned paths. In practice, most autonomous systems are governed by nonlinear dynamics, and the magnitude of inputs is limited either by physical hardware constraints or operational safety considerations. In addition, it is desirable to control the states of these nonlinear systems so that they track the generated paths while adhering to performance constraints, such as rate of convergence, maximum overshoot, and steady-state error. The development of control schemes, capable of managing system uncertainties, nonlinearities, and prescribed performance has been an area of research for several decades, and approaches such as prescribed performance control (PPC) and funnel control (FC) are well explored in the literature [20,21]. The goal of the aforementioned methodologies is to design control laws that guarantee that the tracking error of the system’s output remains within predefined performance bounds. To achieve this, the control effort is designed to increase as the error approaches the performance boundary, which can lead to practically infeasible control magnitudes.

Recent advancements in PPC and FC have proposed robust control schemes to ensure prescribed performance even under input constraints. In work [22], FC under input saturation is studied, and a lower bound for the input saturation level is extracted to ensure adherence to performance constraints. Nonlinear systems of arbitrary relative degree are studied in work [23], where a bang-bang FC methodology is proposed. Neural network approximation is utilized in [24,25] for nonlinear systems, which guarantees prescribed performance under input saturation. Saturation-tolerant PPC is studied in works [26,27] utilizing fuzzy-logic estimators for systems with unknown control directions and time-delay systems, respectively. Approximation-free schemes are utilized in [28,29,30,31,32,33,34]. Tracking with adaptive performance is achieved in [28,30,31], where the prescribed performance specifications adapt to the tracking error in order to accommodate the input constraints. While this ensures the stability of the closed-loop system under input saturation, the prescribed performance guarantees may be relaxed. As a result, undesirable performance can occur in CPP scenarios, such as excessive overshoot, which in turn translates to covering the same area twice. Switching the controller that maintains stability despite input saturation is studied in [33]. Properly adapting the tracking signal to make prescribed performance achievable under control saturation is studied in [32,34], where the performance specifications are correlated to the saturation level. Specifically, in [32], the desired tracking trajectory is modified to remain feasible under input saturation. While this modification achieves the goal of stability, the system’s output is then guaranteed only to track the modified trajectory rather than the originally desired one, which might prove problematic in CPP scenarios. This issue is alleviated in [34], where only the velocity trajectory is modified and the actual position tracking error is guaranteed to be prescribed, provided that proper conditions for the input saturation level are met. A concise comparison of the PPC methodologies under input saturation that are of low complexity (approximation-free) is provided in Table 2. Although simulation experiments are conducted in the aforementioned works, they are often limited to numerical environments. To fully evaluate and refine a control strategy, it is essential to employ high-fidelity simulations that accurately capture the complexities and uncertainties of real-world conditions and to test the interplay between planning and control under realistic scenarios, ensuring robustness and reliability before deployment in physical systems.

### 1.3. Limitations of Existing Methodologies

Implementing a robust framework that integrates path planning and control for AUVs is vital to overcoming challenges in real-world applications like seafloor mapping and underwater debris detection. However, notable limitations remain in the integration of coverage path planning and prescribed performance control approaches.

Regarding coverage path planning, methods that focus on minimizing the number of turns, motivated by the additional energy expenditure required for each turn, often fail to incorporate workspace boundaries and obstacles when connecting the generated back and forth paths. In [19], for instance, the authors consider connecting the sweeping lines using Dubins’ paths that do not explicitly factor in the boundaries of the target area or the presence of obstacles. Consequently, even though such approaches minimize the number of turns in theory, they can yield infeasible paths in cluttered or irregular regions.

On the control side, PPC and FC strategies have demonstrated considerable promise for managing the nonlinear dynamics of AUVs under input saturation constraints. However, most of these approaches still require thorough validation in environments that accurately capture the complexities of real-world underwater operations. Although the authors of [34] provide a rigorous mathematical proof and numerical simulations for Euler-Lagrange systems with input saturation, its effectiveness remains untested in high-fidelity settings that incorporate realistic hydrodynamic effects. Additionally, to ensure efficient coverage, minimal overshoot is essential so that planned paths are not traversed multiple times. Achieving this level of precision typically demands careful parameter tuning and iterative testing, especially when time and energy use are critical concerns in AUV missions.

The challenge of unifying coverage path planning and prescribed performance control becomes apparent when considering external disturbances, sensor uncertainties, actuator limitations, and the need for consistent performance. In practice, the controller must accurately track the planned paths without sacrificing convergence speed or overshoot constraints, while the planning algorithm must produce trajectories that remain feasible under the vehicle’s hardware and dynamic restrictions. Balancing these intertwined requirements calls for a design process where algorithms for path planning and control are developed and thoroughly tested in environments that closely approximate real underwater conditions. By addressing the existing gaps, such as obstacle-aware path connectivity, robust performance under saturation, and precise calibration to limit redundant coverage and mission time, future research can move toward a more comprehensive and validated methodology for autonomous coverage tasks in challenging underwater scenarios.

### 1.4. Contribution

Motivated by the above discussion, in this work, we present a fully integrated framework for path planning and control, validated in a high-fidelity simulation environment using ArduSub SITL, ROS, and Gazebo. For path planning, we build on the approach in [19], introducing a key implementation difference: the costs for traveling between line segments are calculated using an A* algorithm with obstacle avoidance, which considers the actual path length to compute weights for the generalized traveling salesman problem (GTSP). This enhancement provides more realistic path connectivity while ensuring obstacle avoidance. On the control side, we adopt the method from [34], designed for Euler–Lagrange systems, which ensures input saturation constraints are respected without modifying the output error, as in [32], which renders it appropriate for safety and precision critical applications. This choice allows for accurate and reliable tracking of the generated paths. The framework is implemented on a simulated BlueROV2 platform, where the environment closely mirrors real-world conditions.

### 1.5. Organization

The remainder of this paper is organized as follows: Section 2 outlines the problem setting. Section 3 describes the proposed methodology, detailing the path planning algorithm, the reference modification system, and the control design. Section 4 presents the simulation setup and results, demonstrating the effectiveness of the proposed approach in a high-fidelity underwater environment. Finally, Section 5 and Section 6 provide a discussion of the findings, highlight the contributions of this work, and outline directions for future research.

## 2. Problem Formulation

The problem considered in this work is the complete and robust coverage of a predefined underwater 2D region using an autonomous underwater vehicle. The workspace, denoted as $W\subset R^{3}$, is assumed to be a connected, static region with obstacles. The goal is to generate a complete coverage path with minimal turns that avoids obstacles and implements a robust control design that respects the AUV’s dynamic and physical constraints to follow the aforementioned path.

The CPP problem involves dividing W into non-overlapping subregions, generating collision-free paths for the AUV within each subregion, and determining a sequence for visiting these paths. To create these paths, we implement the methodology of [19], and to connect them we incorporate a generalized traveling salesman Problem framework, where the cost of traveling between paths is dynamically calculated using an A* algorithm to account for obstacles and the workspace boundary. This ensures a realistic estimation of the transition costs, essential for optimizing the path length and overall efficiency.

In addition to path planning, the AUV has to track the generated paths with high precision, i.e., minimal overshoot and settling time to avoid overlapping of paths. This requires addressing the nonlinear and potentially partially unmodeled dynamics of the AUV, input saturation, and external disturbances such as undersea currents. A robust prescribed performance control strategy is necessary to ensure the AUV adheres to the planned paths while maintaining stability and performance specifications. To this end, we implement a control scheme designed for Euler–Lagrange systems [34], capable of handling input constraints and adapt it to the problem at hand to ensure accurate waypoint tracking.

The solution is evaluated using a high-fidelity simulation environment that incorporates realistic robot modeling, underwater dynamic environment, hydrodynamic effects and disturbances, providing a comprehensive validation of the proposed framework.

## 3. Materials and Methods

In this section, we provide an overview of the robot dynamics and the methods we implement as well as the standing assumptions.

### 3.1. Underwater Vehicle Kinematics and Dynamics

**Figure 1:**
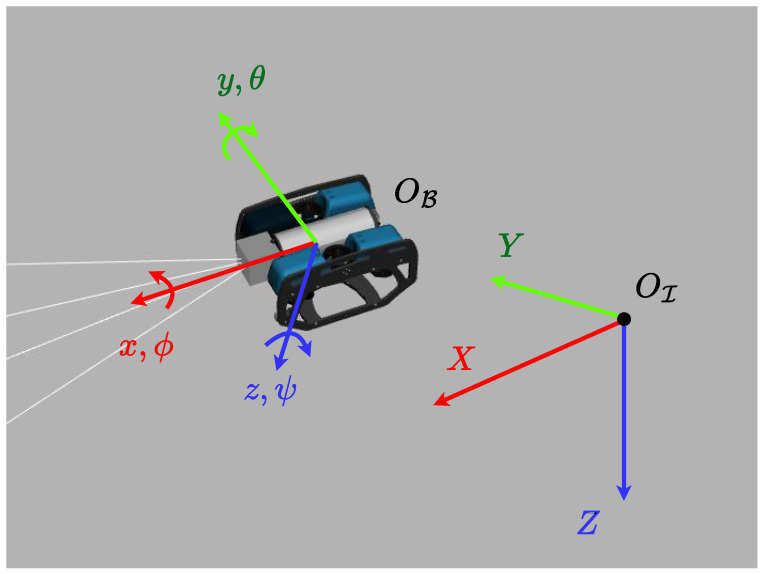
The inertial and body-fixed frames.

The underwater robot we employ in our coverage scheme is the BlueROV2, a commercial robot created by Blue Robotics (https://bluerobotics.com/). We define two frames of reference: a body-fixed frame $O_{B}$ attached to the vehicle’s center of gravity and the inertial frame $O_{I}$, as depicted in Figure 1. We denote by $q={\left[{q_{1}}^{T}\;{q_{2}}^{T}\right]}^{T}\in R^{6}$ the pose vector of the vehicle with respect to the body frame B, where $q_{1}={\left[x\;y\;z\right]}^{T}$ represents the position and $q_{2}={\left[\phi \;\theta \;\psi \right]}^{T}$ represents the orientation of the vehicle. Furthermore, we denote by $v={\left[{v_{1}}^{T}\;{v_{2}}^{T}\right]}^{T}\in R^{6}$ the velocity vector with respect to the fixed-body frame B, where $v_{1}={\left[u\;v\;w\right]}^{T}$ represents the linear and $v_{2}={\left[p\;q\;r\right]}^{T}$ the angular velocity of the vehicle.

The kinematics and dynamics of the vehicle are given by the following equations:

(1) $$\begin{array}{cc} \dot{q}=\; & v+d_{1}\left(t,q\right) \end{array}$$

(2) $$\begin{array}{cc} M\left(q\right)\dot{v}+C\left(q,v\right) & v+d_{2}\left(t,q,v\right)=\mathrm{sat}(\tau ), \end{array}$$

where $M\left(q\right)\in R^{6\times 6}$ represents the inertia matrix, $C\left(q,v\right)\in R^{6}$ is the centrifugal and Coriolis forces vector and vectors $d_{i}\in R^{6},\;i=1,2$ are included to represent bounded external disturbances, unmodeled dynamics, and include gravitational force. Vector $\mathrm{sat}(\tau )={\left[\mathrm{sat}\left(\tau_{1}\right)\cdots \mathrm{sat}\left(\tau_{m}\right)\right]}^{T}\in R^{6}$ is the saturated control input vector of the vehicle, where $\tau ={\left[\tau_{1}\cdots \tau_{6}\right]}^{T}\in R^{6}$ is the total propulsion force/torque generated by the thrusters. We make the following assumptions regarding the model:

**Assumption 1.**  *All matrices and vectors are assumed to be continuous functions with respect to time and locally Lipschitz with respect to the rest of their arguments.*

**Assumption 2.**  *Functions $d_{i},\;i=1,2$ are uniformly bounded with respect to time, thus incorporating the effect of time-dependent bounded disturbances.*

**Assumption 3.**  *The inertia matrix M(q) is diagonal and uniformly positive definite for all $q\in R^{m}$.*

The continuous saturation function sat(·):R→R is defined as

(3) $$\mathrm{sat}\left(\tau_{i}\right)=\left\{\begin{array}{cc} \tau_{i}, & \mathrm{if}\;|\tau_{i}|<\tau_{i,\max}\\ \tau_{i,\max}\cdot \mathrm{sgn}\left(\tau_{i}\right), & \mathrm{otherwise}, \end{array}\right.$$

where $\tau_{i,\max}>0$ is the saturation level for each input component $\tau_{i},\;i=1,\ldots ,6$, and sgn(·) is the signum function.

**Remark 1.**  *The vehicle used in this article is using the “No Heavy Configuration Add-on” thruster configuration [35], which uses 6 thrusters and has no thruster positioned with a pitch component; therefore, it has no pitch control. Thus, we consider that $\tau_{5}\left(t\right)=0,\;\forall t\geq 0$.*

### 3.2. Coverage Path Planning

In this subsection, we introduce the relevant workspace definitions and examine the path planning algorithm. The strategy we follow in the present work is the following: the target area is initially subdivided into smaller sections (subregions). Each subregion is then assigned a coverage orientation based on its generalized width, and a set of line segments aligned with this orientation is generated to ensure complete coverage. Finally, a combination of a generalized traveling salesman problem and an A* algorithm is employed to link these line segments into a single continuous path.

In the literature [17,19], minimizing the total number of turns is widely regarded as the optimality criterion utilized for an efficient coverage solution. Because the number of turns is directly linked to each subregion’s generalized width (measured in the sweep direction), our decomposition approach seeks to minimize these widths. Moreover, to enhance overall optimality, the generalized traveling salesman algorithm employed to connect the line segments prioritizes minimizing path lengths while avoiding obstacles, further expanding the optimality and enhancing the applicability of the algorithm.

**Remark 2.**  *In work [19], the authors refer to the “generalized width” as “altitude”. However, we opt to call it “generalized width” to avoid unnecessary confusion between the notion of polygon altitude and the distance between the robot is operating at and the seafloor, which is often called altitude in the AUV literature.*

#### 3.2.1. Minimum Turn Decomposition

We assume that the robot is seeking to completely cover an underwater region represented by a polygonal workspace $W\subset R^{3}$. More specifically, we study a top–down cross-section of *W*, for which we make the following assumptions:

**Assumption 4.**  *The CPP algorithm is performed on a top–down cross-section $P\subset R^{2}$ of W that corresponds to a fixed distance from the seabed.*

**Figure 2:**
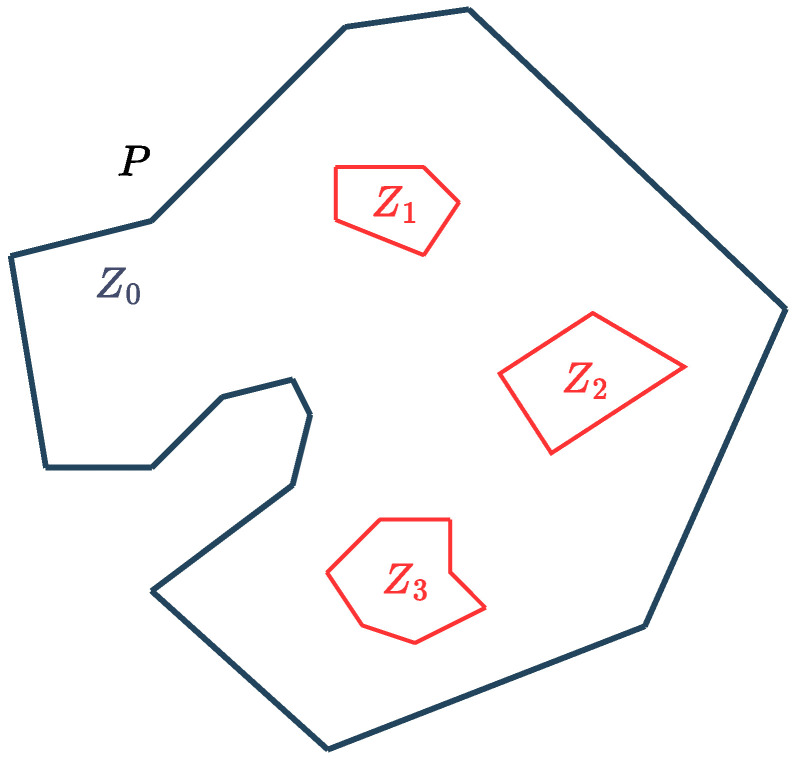
Cross-section P of the underwater workspace, comprising an exterior polygonal boundary $Z_{0}$ and interior polygonal obstacles $\left\{Z_{1},\cdots ,Z_{M}\right\}$.

**Assumption 5.**  *$P=\{Z_{0},\cdots ,Z_{M}\}$ is a 2D polygon, defined by an exterior simple polygon $Z_{0}$ that represents the workspace boundary, and interior simple polygons $\left\{Z_{1},\cdots ,Z_{M}\right\}$ that represent obstacles in the workspace, as shown in Figure 2.*

The reason we focus our work on a 2D environment instead of the full 3D workspace is because many underwater applications, such as surveying, garbage mapping and underwater cable inspection, are efficiently operating at a specific depth level or with specific vertical constraints, reducing the CPP requirements to a 2D space.

**Figure 3:**
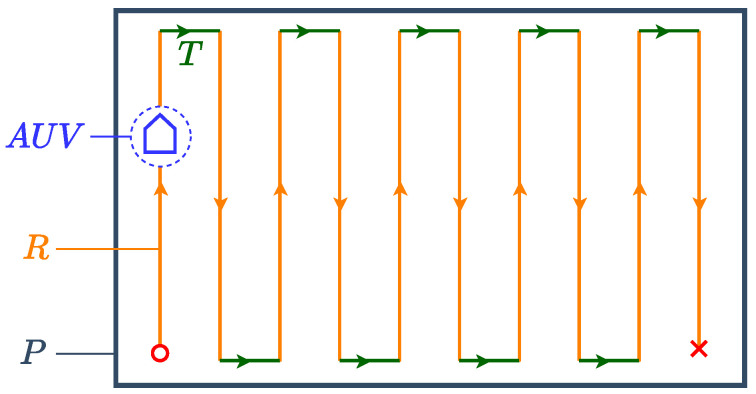
Complete coverage of polygon P with straight polygonal segments *R* connected by turns *T*.

The complete coverage path of the polygonal workspace P is a so-called polygonal sweeping path comprising two types of path segments. The first type is a set of parallel straight line segments *R* that cover the largest portion of the workspace. The second type is a set of arbitrarily shaped paths *T*, called turns, each of which connects two straight line segments *R*. An example coverage path is shown in Figure 3, wherein turns *T* are straight lines. We assume that the robot has a circular footprint and that two successive parallel segments *R* are distanced one footprint diameter from each other to avoid coverage redundancies.

Since performing turn *T* requires the acceleration and deceleration of the underwater robot, as noted in the literature [17,19], we posit that an energy-efficient CPP algorithm seeks to minimize the number of turns |T|. Minimizing the number of turns |T| is equivalent to minimizing the number of straight paths |R| required for coverage, since every two straight paths *R* are connected by at most one turn *T* as can be seen in Figure 3. Therefore, as the first step of out CPP methodology, we seek to partition P into polygonal subregions, such that the number of straight sweeping paths |R| required for complete coverage is minimized.

**Figure 4:**
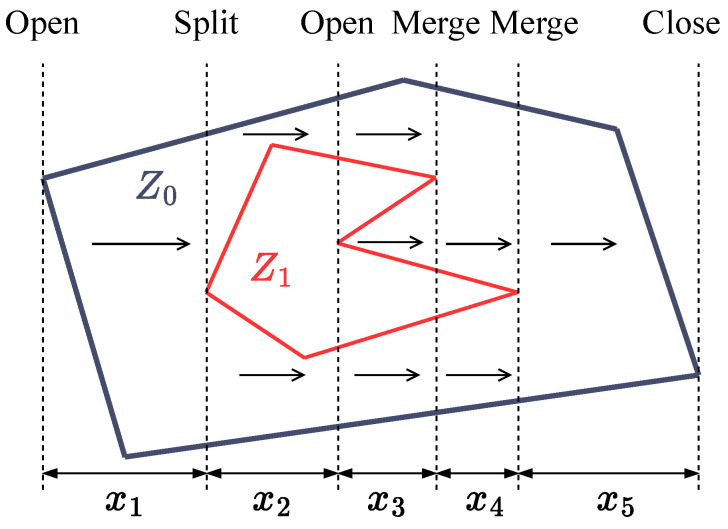
Example process of calculating the generalized width of non-convex polygon $\left\{Z_{0},Z_{1}\right\}$ at angle $0^{\circ }$. The generalized width is equal to $x_{1}+2x_{2}+3x_{3}+2x_{4}+x_{5}$. The algorithm is based on the “Open”, “Close”, “Split”, and “Merge” events described in [17].

For optimizing the workspace decomposition, we follow the methodology introduced in [19], which we briefly describe. First, a measure of the generalized width of a general non-convex polygon is defined for a given direction. An example polygon and its corresponding generalized width at a given direction is shown in Figure 4.

The authors observe that the sweeping direction for which the number of straight path segments |R| in a polygon is minimized is related to the direction of minimum generalized width of the polygon. With this in mind, they define a cost function for a given partition of P as follows:

(4) $$\sum_{i=1}^{k}a^{*}\left(P_{i}\right)$$

where $a^{*}$ is the minimum generalized width of a polygon and $P_{i}$ is a polygonal subregion of partition $D=\{P_{1},P_{2},\cdots ,P_{k}\}$, where ${⋃}_{i=1}^{k}P_{i}=P$.

**Figure 5:**
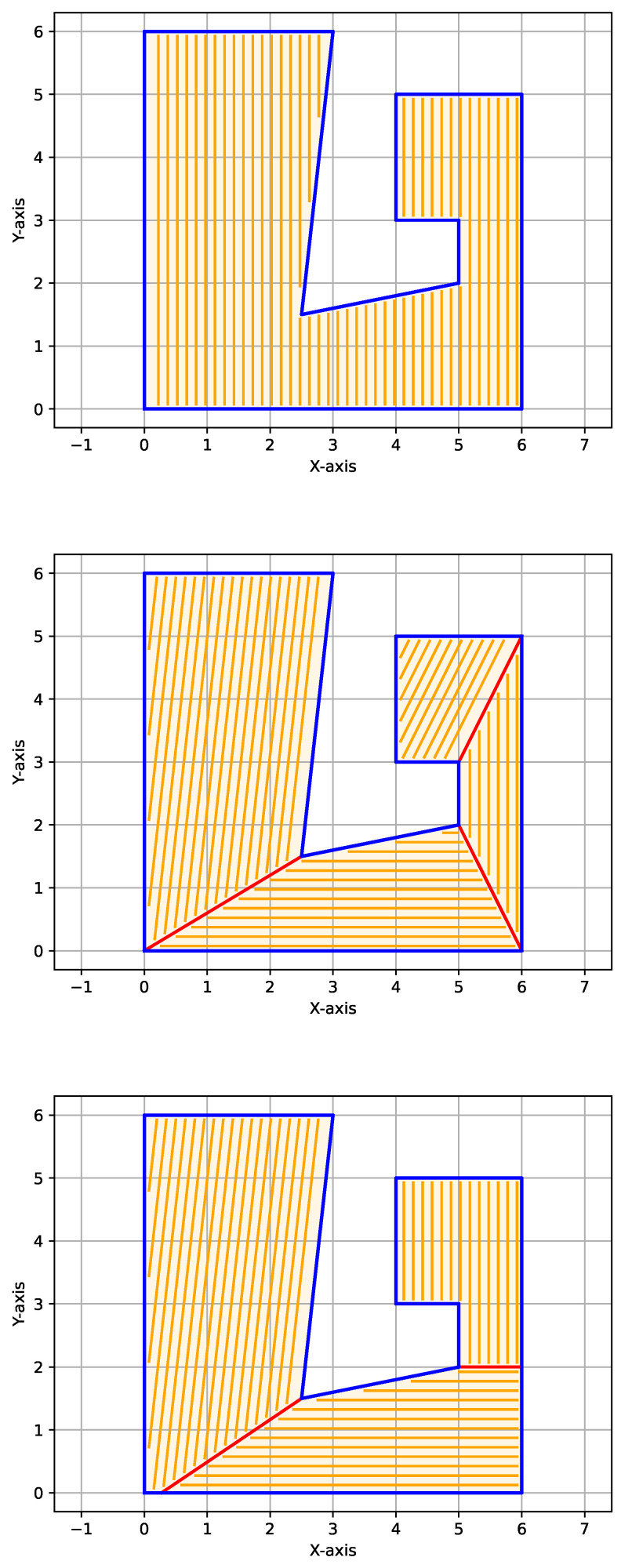
Demonstration of the minimum turns decomposition algorithm acting on a non-convex polygon. No decomposition |R|=49 (**top**), initial convex decomposition |R|=47 (**middle**) and optimal decomposition |R|=44 (**bottom**).

P is initially decomposed into a set of convex sub-polygons D, following the convex decomposition algorithm introduced in [36]. Then, each cut formed by the initial partition D is successively re-optimized, such that the cost (4) of the current partition is less than that of the previous. This procedure is executed iteratively until it converges to a partition that minimizes the cost. Finally, by filling each sub-polygon of the optimal decomposition with its corresponding straight sweep segments *R* according to the direction of minimum altitude, we place the minimum number of straight segments required for the complete coverage of P. Figure 5 shows a demonstration of the minimum turn decomposition algorithm on a non-convex polygon.

#### 3.2.2. Minimum Cost Path

**Figure 6:**
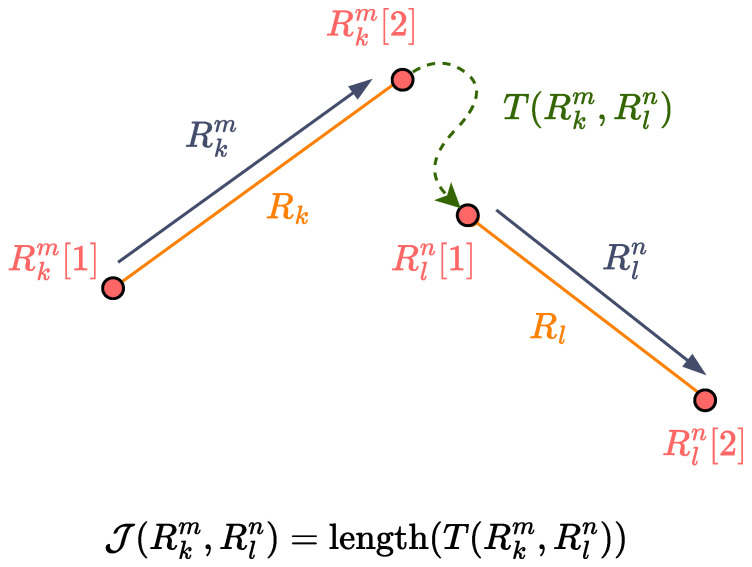
Calculation of transition cost $J(R_{k}^{m},R_{l}^{n})$ of the turn segment $T(R_{k}^{m},R_{l}^{n})$ from $R_{k}^{m}$ to $R_{l}^{n}$.

After generating the minimum amount of straight segments *R* for each polygonal subregion in P, we have to appropriately connect them with turn segments *T* in order to complete the coverage path. Note that each straight segment path $R_{k}\in R$ has two possible traversal directions, denoted as $R_{k}^{1},R_{k}^{2}$. We define $J(R_{k}^{m},R_{l}^{n})$ as the transition cost from segment $R_{k}$ in direction *m* to segment $R_{l}$ in direction *n*, where m,n∈{1,2} and k,l∈{1,⋯,M} with k≠l and M=|R|. Here, $J(R_{k}^{m},R_{l}^{n})$ is taken to be the arc length of the turn segment $T(R_{k}^{m},R_{l}^{n})$, which connects the end point $R_{k}^{m}\left[2\right]$ of $R_{k}^{m}$ and the start point $R_{l}^{n}\left[1\right]$ of $R_{l}^{n}$, as illustrated in Figure 6. Calculating the cost J for each pair $R_{k}^{1},R_{k}^{2}$ enables the construction of a complete graph G=(V,E,w), following [19]. Here, each vertex v∈V represents a straight path direction $R_{k}^{m}$ for m∈{1,2} and k∈{1,⋯M}, each edge e=(v,z)∈E represents a transition between vertices *v* and *z*, and weights *w* represent the respective transition costs between vertices. Because each edge is a straight path direction, it holds that |V|=2|R|. Finally, *V* is partitioned into clusters of directions $\left\{R_{k}^{1},R_{k}^{2}\right\}$ where k={1,⋯,M}. The graph with the clusters forms a GTSP instance, whose solution leads to the complete coverage path.

**Figure 7:**
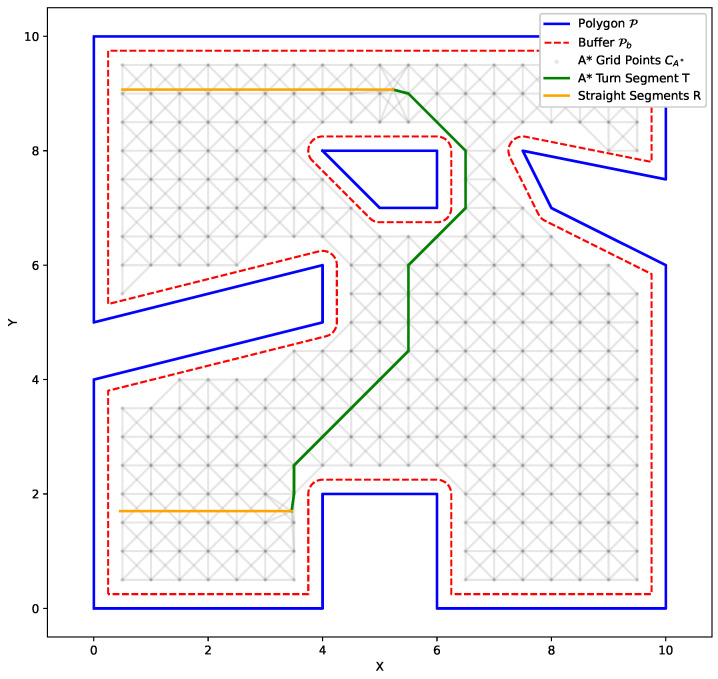
Calculation of the transition cost between two straight segments R inside a uniform A* grid with a resolution of 0.5 and robot footprint size of 0.5. The transition cost is equal to the length of the turn T produced by A* and is calculated to be 9.238.

To calculate transition costs $J(R_{k}^{m},R_{l}^{n})$ of the GTSP instance, we employ a conventional A* algorithm, which enables robust obstacle avoidance and a fully connected graph. First, we construct a grid from the geometry of P, as described in Algorithm 1. Then, we use A* to compute the shortest path between the end point $p_{i}=R_{k}^{m}\left[2\right]$ of directional segment $R_{k}^{m}$ and the start point $p_{j}=R_{l}^{n}\left[1\right]$ of $R_{l}^{n}$, where i,j∈N=4|R|,i≠j. Then, we calculate the distance traveled following the path produced, and we assign that distance as the cost of the transition. As such, in order to complete graph *G*, we need to compute the transition cost matrix *W*. The calculation procedure is described in Algorithm 2. An example transition cost calculation inside a uniform A* grid is illustrated in Figure 7.
**Algorithm 1** A* Grid Construction1:**Input:**$P=\{Z_{0},Z_{1},\cdots ,Z_{M}\}$, straight segments R, robot footprint diameter *d*2:$Z_{b0}\leftarrow Z_{0}$ buffered inwards by d/23:**for** each inner polygon $Z_{i}$, i=1,2,⋯,M **do**4:    $Z_{bi}\leftarrow Z_{i}$ buffered outwards by d/25:**end for**6:$P_{b}\leftarrow \left\{Z_{b0},Z_{b1},\cdots ,Z_{bm}\right\}$7:$C_{A*}\leftarrow$ set of points produced by any mesh generation algorithm of choice on $P_{b}$8:**for** each $R_{k}\in R$ **do**9:    add points $R_{k}\left[1\right]$, $R_{k}\left[2\right]$ to $C_{A*}$10:**end for**11:**Return** Grid $C_{A*}$

**Algorithm 2** GTSP-A* Transition Cost Matrix Calculation
1:**Input:** Straight segments *R*, A* grid2:Add start and end points *p* of $R_{k}^{m}$ in grid and connect with neighbors3:**for** each end point $p_{i}=R_{k}^{m}\left[2\right]\in p$ **do**4:    **for** each start point $p_{j}=R_{l}^{n}\left[1\right]\in p$ **do**5:        Generate A* path $T_{ij}=T\left(R_{k}^{m},R_{l}^{n}\right)$ between $p_{i}$ and $p_{j}$6:        $J_{ij}\leftarrow$ distance traveled from end point $p_{i}$ to start point $p_{j}$ following $T_{ij}$7:        $W\left[i\right]\left[j\right]\leftarrow J_{ij}$8:    **end for**9:
**end for**
10:**Return** Transition Cost Matrix *W*


### 3.3. Control Design

The controller design involves two modules in the spirit of [34]. First, a reference modification module is designed that alters the virtual velocity references to render them feasible under input saturation. Then, a prescribed performance controller is designed employing the modified velocity reference in the velocity error calculation.

#### 3.3.1. Reference Modification Module

We design a module that distorts velocity reference signals to make them reachable under input constraints, as dictated by vector $v_{\mathrm{mod}}={\left[v_{\mathrm{mod},1}\cdots v_{\mathrm{mod},6}\right]}^{T}\in R^{6}$ with the following dynamics:

(5) $${\dot{v}}_{\mathrm{mod},i}\left(t\right)=-\beta_{i}v_{\mathrm{mod},i}\left(t\right)+\Delta \tau_{i},\;v_{\mathrm{mod},i}\left(0\right)=0,$$

where $\Delta \tau_{i}=\mathrm{sat}\left(\tau_{i}\right)-\tau_{i}$ and $\beta_{i}>0$ are design constants for all i=1,…,6.

#### 3.3.2. Waypoint Tracking Control Design

The control design follows a two-step procedure:

*Step 1:* Definition of the normalized position error variable vector $\xi_{p}:={\left[\xi_{p,1}\cdots \xi_{p,6}\right]}^{T}$ as:

(6) $$\begin{array}{cc} \xi_{p}\left(t\right) & =R_{p}^{-1}\left(t\right)\left(q\left(t\right)-q_{d}\right), \end{array}$$

where $R_{p}\left(t\right)=\mathrm{diag}\left(\rho_{p,1}\left(t\right),\ldots ,\rho_{p,6}\left(t\right)\right)$ and $q_{d}$ is the target waypoint with respect to the local frame B. To incorporate the transient and steady-state position error performance requirements, we utilize the prescribed performance functions $\rho_{p,i}$, which are considered to be continuously differentiable, positive, decreasing, have a constant positive limit as time tends to infinity, and satisfy $\rho_{p,i}\left(0\right)>\left|q_{p,i}\left(0\right)-q_{d,i}\right|$. An example for the definition of the functions is $\rho_{p,i}\left(t\right):=\left(\rho_{p,i}^{0}-\rho_{p,i}^{\infty }\right)e^{-\lambda_{p,i}t}+\rho_{p,i}^{\infty }$, where $\lambda_{p,i}>0$, $\rho_{p,i}^{0}>\left|q_{p,i}\left(0\right)-q_{d,i}\right|$ and $\rho_{p,i}^{\infty }>0$ are designable parameters, for all i=1,…,6. Then, we design the virtual control reference signal $v_{d}:={\left[v_{d,1}\cdots v_{d,6}\right]}^{T}$ as

(7) $$\begin{array}{c} v_{d,i}\left(t\right)=-k_{p,i}T\left(\xi_{p,i}\right),\;i=1,\ldots ,6, \end{array}$$

where $k_{p,i}$ are positive design constants and T:(−1,1)→R is a strictly increasing, continuously differentiable function, satisfying $\lim_{x\to -1^{+}}T\left(x\right)=-\infty$ and $\lim_{x\to 1^{-}}T\left(x\right)=\infty$.

*Step 2:* Definition of the normalized velocity error variable vector $\xi_{v}:={\left[\xi_{v,1}\cdots \xi_{v,6}\right]}^{T}$ as

(8) $$\xi_{v}\left(t\right)=R_{v}^{-1}\left(t\right)\left(v\left(t\right)-v_{d}\left(t\right)-v_{\mathrm{mod}}\left(t\right)\right)$$

where $R_{v}\left(t\right)=\mathrm{diag}\left(\rho_{v,1}\left(t\right),\ldots ,\rho_{v,6}\left(t\right)\right)$. To incorporate the transient and steady-state modified velocity error performance requirements, we utilize the prescribed performance functions $\rho_{v,i}$, which are considered to be continuously differentiable, positive, decreasing, having a constant positive limit as time tends to infinity, and satisfy $\rho_{v,i}\left(0\right)>\left|v_{i}\left(0\right)\right|$. An example for the definition of the functions is $\rho_{v,i}\left(t\right):=\left(\rho_{v,i}^{0}-\rho_{v,i}^{\infty }\right)e^{-\lambda_{v,i}t}+\rho_{v,i}^{\infty }$, where $\lambda_{v,i}>0$, $\rho_{v,i}^{0}>\left|v_{i}\left(0\right)\right|$ and $\rho_{v,i}^{\infty }>0$ are designable parameters for all i=1,…,6. Then, we design the control law components as

(9) $$\tau_{i}\left(t\right)=-k_{v,i}\frac{1}{\rho_{v,i}\left(t\right)}\frac{\partial T(\xi_{v,i})}{\partial \xi_{v,i}}T\left(\xi_{v,i}\right),$$

where $k_{v,i}$ are positive design constants for every i=1,…,6.

Here, we recite Theorem 1 of [34] for completeness of presentation.

**Theorem 1.**  *We consider an Euler–Lagrange system described by (1) and (2) and Assumption 3. We define $F_{v,i}\left(q,v,t\right):=\left|{\left(C\bar{v}\right)}_{i}\right|+\left|d_{2,i}\left(q,v,t\right)\right|+m_{i}\left|{\bar{\dot{v}}}_{d,i}\right|+m_{i}\left|{\dot{\rho }}_{v,i}\left(t\right)\right|$ and sets ${\bar{S}}_{v}:={\bar{S}}_{p}\times \left\{v||v_{i}\left(t\right)|\leq {\bar{v}}_{i},i=1,\ldots ,6\right\}$ with ${\bar{S}}_{p}:=\left\{q||q_{i}\left(t\right)|\leq {\bar{q}}_{i},i=1,\ldots ,6\right\}$, where ${\left(C\bar{v}\right)}_{i},{\bar{\dot{v}}}_{d,i},{\bar{v}}_{i}$ and ${\bar{q}}_{i}$ are constants defined in [34] that depend on the system model parameters.*

*If Control Law (9) is employed and the input constraints satisfy the following conditions,*

(10) $$\tau_{i,\max}\geq \max\left\{\tau_{i,\max}\left(0\right),\underset{t\geq 0}{\sup}\max_{\left(q,v\right)\in {\bar{S}}_{v}}F_{v,i}\left(q,v,t\right)\right\},$$

*for every i=1,…,6, then all closed-loop signals remain bounded and the generalized position tracks the desired trajectory with prescribed performance in the sense that*

(11) $$\begin{array}{c} |q_{i}\left(t\right)-q_{d,i}|<\rho_{p,i}\left(t\right). \end{array}$$

*In addition, velocity tracks the modified virtual velocity with prescribed performance in the sense that*

(12) $$|v_{i}\left(t\right)-v_{d,i}\left(t\right)-v_{\mathrm{mod},i}\left(t\right)|<\rho_{v,i}\left(t\right),$$

*for all t≥0 and i=1,…,m.*

**Remark 3.**  *Although specific knowledge of the system parameters is not strictly required for the control design, having more precise parameter identification would simplify the accurate calculation of the control limits (see (10)). In the absence of a full system identification, we leverage simulation-based trial and error to determine feasible and realistic saturation limits, thus laying a solid foundation for eventual real-world implementation.*

## 4. Results

In this section, we present the exact materials and methodologies as well as the parameters involved in the implementation of the aforementioned scheme.

### 4.1. Simulation Software

The proposed framework was implemented using a combination of tools and software platforms to simulate and validate the path planning and control strategies. The control algorithm and path planner were developed in Python, leveraging its flexibility and extensive libraries for mathematical computation and algorithm design. The entire system was integrated into the Robot Operating System (ROS), a widely used framework for robotic applications, ensuring modularity and communication between components.

To simulate the underwater environment and validate the proposed methods, Gazebo was employed as the high-fidelity simulation platform. The ArduSub SITL was utilized to emulate the BlueROV2’s onboard control architecture, ensuring realistic hydrodynamic behaviors and sensor feedback. Additionally, the ROS package “bluerov_ros_playground” was used to interface the simulated BlueROV2 with the ROS environment, providingaccess to virtual actuators. For testing the controller in this simulation, we leveraged readily available velocity and position odometry data. This integration enabled seamless testing of the control and planning algorithms under realistic underwater conditions.

### 4.2. Coverage Path Planning

We implement the decomposition algorithms developed in [19] and our own algorithm for transition cost computation in Python 3.11, along with the libraries Shapely [37] and Py2D [38]. For the solution of the GTSP instance, we leveraged the solver GLKH [39,40].

**Figure 8:**
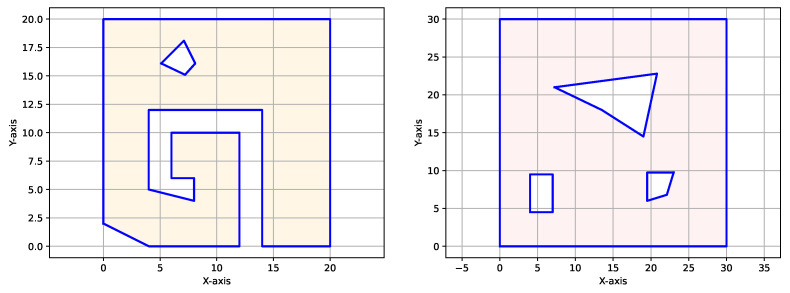
Two polygonal workspaces used to demonstrate the GTSP-A* CPP algorithm. The left-side workspace focuses on the non-convexity of the exterior boundary, while the right-side workspace focuses on the presence of obstacles.

We demonstrate our methodology on the two polygonal workspaces shown in Figure 8. For the CPP problem, we assume a footprint diameter of 1m for BlueROV2, which is greater than its length of 0.457 m. This choice of footprint diameter was made to account for the fact that a coverage footprint usually represents the field of view of a sensor rather than physical dimensions.

First, both workspaces are decomposed into sub-polygons that minimize the number of turns for coverage, and then each sub-polygon is filled with the minimum amount of straight sweep segments. Then, a grid is generated with respect to each polygonal workspace geometry using Algorithm 1 in order to facilitate the A* path generation. The grid is uniform and structured, with a resolution of 0.5 m for the left workspace in Figure 8, and 0.6 m for the right workspace. Here, we construct the grid so that each grid point has eight points as neighbors, allowing for horizontal, vertical, and diagonal movement between nodes, as can be seen in Figure 7. Finally, the rest of Algorithm 2 is executed in order to compute the costs of the turns between the straight sweep segments, leading to the generation of the complete coverage path.

**Figure 9:**
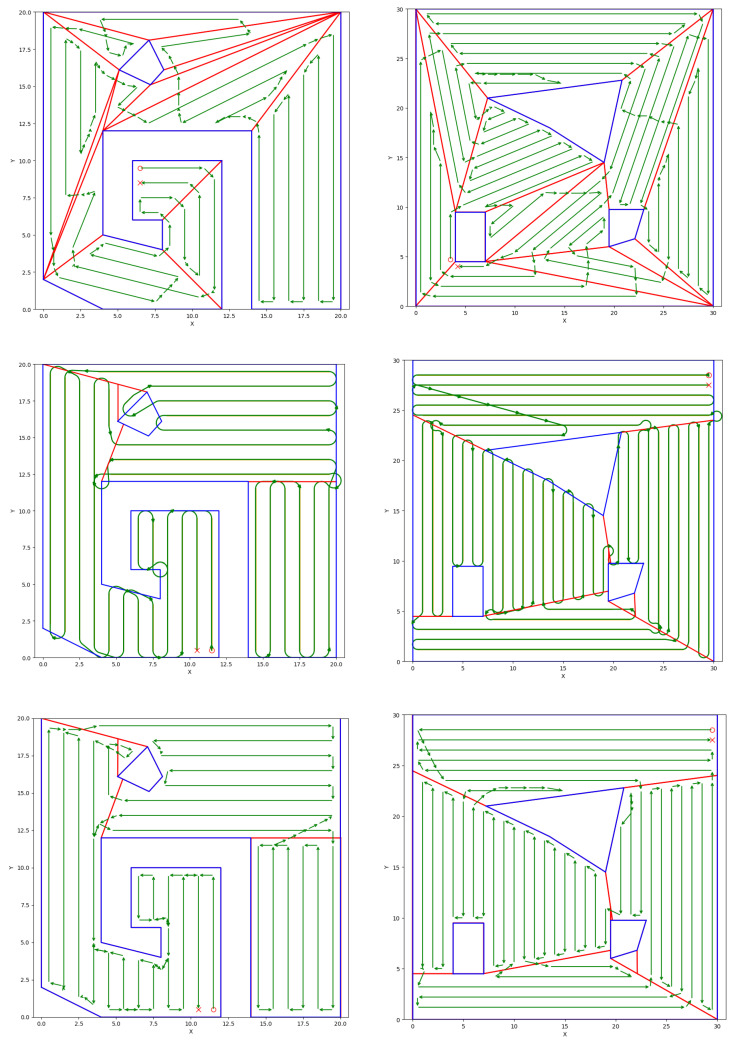
**First Row**: Greedy convex partition and A* transition cost. **Second Row**: Minimum turn partition and Dubins’ transition cost. **Third Row**: Minimum turn partition and A* transition cost. “O” marks the entry point while “X” marks the finishing point of the complete coverage paths.

In Figure 9, we provide comparison between the results of the proposed algorithm and two different scenarios. In the first scenario presented in the first row, we utilize a “greedy” convex decomposition algorithm to construct the straight segments *R*, use our GTSP-A* scheme to form the complete coverage path, and calculate the transition costs. In the second scenario presented in the second row, we employ the methodology used in [19]. We perform the turn-minimizing partition and utilize Dubins’ vehicle to construct turns *T* and formulate the corresponding transition costs. Finally, in the third row, we demonstrate the results of our implementation.

It is evident from Figure 9 that the coverage paths produced from all methodologies indeed cover almost the entirety of the polygonal workspaces. Additionally, it is worth noting that because all three methods solve a GTSP problem to construct the coverage path, the entry points and finishing points of the coverage paths lie adjacent to each other. This is the case due to the fact that the GLKH implementation produces a looping path. This is in line with our use case, since we want BlueROV2 to return to its initial docking position after performing complete coverage of the target area.

The differences in the performance of each methodology can also be observed from Figure 9. The greedy convex decomposition algorithm from the first row produces 30 and 51 paths for the left- and right-side workspaces, respectively. This is in contrast to the 29 and 41 paths produced by the minimum turns decomposition algorithm for the left- and right-side workspaces, respectively, from the second and third rows. This comparison shows that depending on the geometrical characteristics of the polygonal workspace, as well as the size of the robot’s footprint, the differences in the produced number of turns between a naive convex decomposition and an optimized, turn-minimizing workspace partition can vary from marginal to substantial. Comparing the results from the second row employing Dubins’ transition costs and the third row which utilizes A* to compute the transition costs, it becomes apparent that the GTSP-A* methodology is better equipped to handle the presence of obstacles and non-convex workspaces.

### 4.3. Controller Design and Parameter Selection

Since our objective is to achieve 2D area coverage, the most critical modes of motion are the horizontal surge and sway, corresponding to movement in the *x* and *y* directions. While the BlueROV2 can also be commanded in vertical and yaw degrees of freedom [41], engaging these additional controls would introduce unnecessary complexity and increase the risk of disturbances. By focusing on horizontal planar motion, we simplify trajectory planning and control, reduce computational overhead, and maintain more stable and predictable vehicle behavior for achieving consistent map coverage. Consequently, we set the remaining thrusters to neutral to avoid coupling effects that do not contribute to our planar coverage goals. The controller is set to receive waypoints $q_{d}$ from the path planner, initialize the prescribed performance variables, and track the first waypoint. After satisfactory tracking is achieved, the controller is reinitialized and the next waypoint is targeted. The local position error is defined as $q\left(t\right)-q_{d}$.

The controller parameters are chosen as follows:

Modification Module Parameter $\beta_{i}$: This parameter controls how fast the modification signal $v_{\mathrm{mod},i}$ adapts to changes in $\Delta \tau_{i}$, which represents the deviation of the desired control effort $\tau_{i}$ from the saturated $\mathrm{sat}\tau_{i}$. A larger $\beta_{i}>0$ causes faster adjustments to the velocity reference whenever the control input becomes saturate, adding to the controller’s effort. In our simulation scenario, the reference modification module parameters are chosen as $\beta_{i}=0.1,\;i=1,2$.

Prescribed Performance Functions for Position $\rho_{p,i}\left(t\right)$: These directly bind the position-tracking error. In our simulation scenario, we choose $\rho_{p,i}\left(t\right)$ as $\rho_{p,i}\left(t\right):=\left(\rho_{p,i}^{0}-\rho_{p,i}^{\infty }\right)e^{-\lambda_{p,i}t}+\rho_{p,i}^{\infty }$ for all i=1,2. The steady-state position error allowance is set by $\rho_{p,i}^{\infty }$, while the allowable initial deviation is captured by $\rho_{p,i}^{0}$. The parameter $\lambda_{p,i}$ dictates the rate of exponential decay, thereby defining how quickly the system converges to its steady state. Larger $\lambda_{p,i}$ values shorten convergence time but may increase control effort. These parameters are set as $\lambda_{p,i}=0.15$, $\rho_{p,i}^{0}=2$ and $\rho_{p,i}^{\infty }=0.1$ for all i=1,2.

Prescribed Performance Functions for Velocity $\rho_{v,i}\left(t\right)$: These bind the modified velocity-tracking error and must remain consistent with the actuator’s saturation levels (Equation (10)). In our simulation scenario, we choose $\rho_{v,i}\left(t\right)$ as $\rho_{v,i}\left(t\right):=\left(\rho_{v,i}^{0}-\rho_{v,i}^{\infty }\right)e^{-\lambda_{v,i}t}+\rho_{v,i}^{\infty }$ for all i=1,2. The steady-state modified velocity error allowance is set by $\rho_{v,i}^{\infty }$, while the allowable initial deviation is captured by $\rho_{v,i}^{0}$. The parameter $\lambda_{v,i}$ dictates the rate of exponential decay, thereby defining how quickly the system’s modified velocity converges to its steady state. Larger $\lambda_{v,i}$ values shorten convergence time but may increase control effort and lead the controller to saturation, impacting the modification module and in turn modifying the velocity reference. These parameters are set as $\lambda_{v,i}=0.05$, $\rho_{v,i}^{0}=5$ and $\rho_{v,i}^{\infty }=0.2$ i=1,2.

Controller Gains $k_{p,i}$ and $k_{v,i}$: These gains scale the overall control action. As in many PPC frameworks, $k_{p,i}$ and $k_{v,i}$ are associated with the size of the bound of the desired velocity reference and the control effort, respectively. In the simulation scenario, we opt for $k_{p,i}=1$ and $k_{v,i}=1$ for every i=1,2.

Finally, function T(·) is chosen as $T\left(\cdot \right)=\ln\frac{1+(\cdot )}{1-(\cdot )}$, and therefore

$$\begin{array}{c} v_{d,i}\left(t\right)=-k_{p,i}\ln\left(\frac{1+\xi_{p,i}}{1-\xi_{p,i}}\right),\;i=1,2. \end{array}$$

Then, the control law components are designed as

$$\tau_{i}\left(t\right)=-k_{v,i}\frac{1}{\rho_{v,i}\left(t\right)}\frac{2}{\left(1-\xi_{v,i}^{2}\right)}\ln\left(\frac{1+\xi_{v,i}}{1-\xi_{v,i}}\right).$$

The saturation limits are chosen as $\tau_{1,\max}=0.3$ and $\tau_{1,\max}=0.5$.

**Remark 4.**  *In practice, the above parameters are determined through iterative simulations in a high-fidelity environment (ArduSub SITL, ROS, and Gazebo). While some trial and error is involved, each parameter directly corresponds to a distinct aspect of performance and feasibility. For instance, if excessive overshoot is observed, translating to covering the same region multiple times, either $\rho_{p,i}^{0}$ or $\lambda_{p,i}$, can be decreased to impose stricter initial performance or slower convergence, respectively, thus lowering the risk of overshoot. An iterative simulation procedure is also followed to determine how strict the performance bounds can be according to Equation (10). Specifically, one might start by fixing the desired input saturation levels, then fine-tune the performance functions via trial and error until an acceptable balance is reached. Alternatively, by fixing the prescribed performance functions first, the minimum saturation levels needed to meet these performance objectives can be derived. Ultimately, the chosen parameters can be tailored to meet specific mission goals, such as faster coverage or tighter tracking precision. Due to the fact that this process can be involved and time-intensive, a potential direction for future work might be the development of an auto-tuning mechanism to streamline and optimize parameter selection.*

### 4.4. Simulation Results

**Figure 10:**
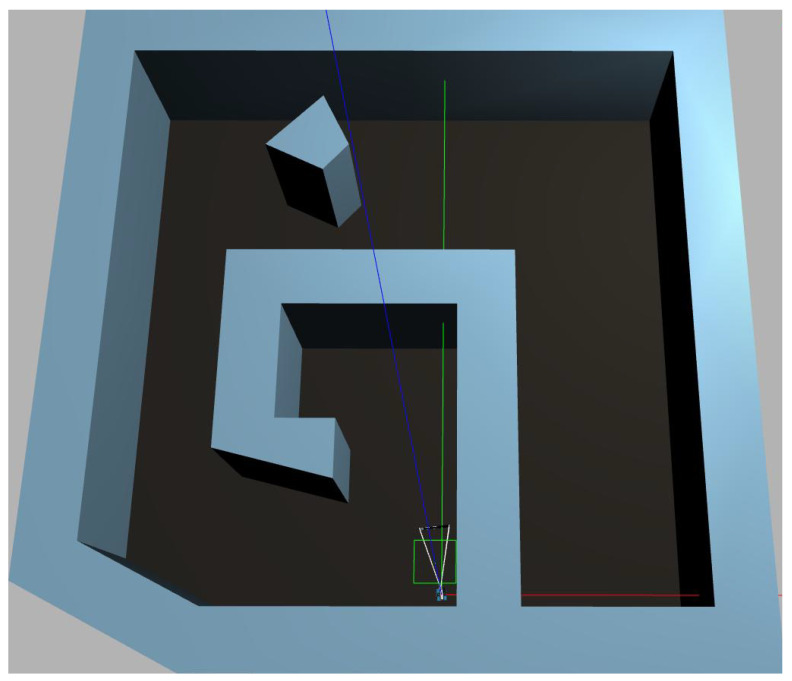
Top view of polygonal workspace and BlueROV2 inside simulated Gazebo world.

The CPP methodology designed in Section 4.2 along with the controller designed in Section 4.3 are combined, and the results are demonstrated in a simulated Gazebo workspace. The simulated workspace is equivalent to the left-side polygonal workspace constructed in Figure 8 and is equipped with the package “freefloating-gazebo” [42] that simulates underwater buoyancy and viscous forces. The simulated workspace is shown in Figure 10. The results of the proposed controller and path planner are demonstrated in the accompanying video [43].

## 5. Discussion

The proposed framework for integrating coverage path planning and robust control for autonomous underwater vehicles demonstrated promising results in efficiently covering 2D areas. By employing a cost-minimizing decomposition algorithm and leveraging an A* search for path connectivity, the approach generates coverage paths that reduce unnecessary turning and alleviate obstacle avoidance challenges. The prescribed performance control scheme ensures that the vehicle accurately tracks the planned paths without exceeding input saturation limits. High-fidelity simulations in Gazebo and ArduSub SITL provide a realistic testing environment, incorporating hydrodynamic effects and disturbances, thereby highlighting both the robustness and feasibility of the proposed method.

Notwithstanding, several limitations emerge. First, in real-world applications, sensing constraints may undermine formal assumptions about the localization of the robot. The BlueROV2 does not possess full sensing capabilities and localization error risk, causing the vehicle to miss the coverage path. A simple mediation measure involves upgrading the sensing equipment of the BlueROV2 and utilizing sensor fusion techniques. Additionally, in scenarios with dynamic obstacles, e.g., moving marine life or other underwater vehicles, a static preplanned path may be insufficient. Real-time obstacle detection and online re-planning are required to adaptively maintain coverage while avoiding collisions. These considerations become even more critical when scaling to larger domains, where increased uncertainty and the higher likelihood of encountering unforeseen obstacles demand robust path planning and updating.

Furthermore, extending this coverage framework from planar to fully three-dimensional environments introduces significant challenges. The coverage methodology needs to be properly adapted for addressing 3D regions. Moreover, developing a sensor fusion technique for accurate 3D localization becomes paramount, as depth and orientation uncertainties can severely impact coverage accuracy. Robotic motion in the water also requires robust control of pitch and roll; therefore, the whole version of the presented controller is to be employed. Additionally, 3D obstacle detection and avoidance involve extra degrees of freedom and potentially faster re-planning to evade moving objects that may approach from above or below. Addressing these new dimensions of 3D partitioning, sensor requirements, control complexity, and dynamic obstacle handling is crucial for extending the results of the present work to 3D environment, and it would pave the way for more comprehensive underwater coverage missions.

Finally, the use of model-free controllers necessitates careful parameter tuning to ensure adequate path tracking. Though manageable in simulation, this process can become more complex when operating over extensively large areas. While the associated tuning effort proved manageable in practice, a conservative choice of the prescribed performance functions is often required to guarantee stability, potentially prolonging total mission duration. Future research could focus on developing automatic tuning strategies to streamline this process without compromising safety.

In sum, while the presented framework demonstrates robust and effective coverage capabilities in simulation environments that approximate real-world conditions, addressing these localization constraints and controller tuning remains essential for scaling up to larger domains and further strengthening the method’s reliability for real-world deployments. By enhancing sensing and localization to address environmental uncertainties, integrating real-time path adaptation to manage dynamic obstacles, and refining controller tuning, the method can maintain its efficiency and robustness at larger scales. These improvements would extend its scalability and resilience, thereby further advancing the viability of autonomous underwater coverage operations in practice.

## 6. Conclusions

This study presented a comprehensive framework combining coverage path planning and robust control for autonomous underwater vehicles. By leveraging a cost-effective decomposition algorithm, obstacle-aware path connectivity, and prescribed performance control, the framework ensured complete and efficient 2D area coverage while maintaining robustness to disturbances and respecting input constraints. The results highlight the potential of the proposed approach for practical applications such as underwater mapping, debris detection, and infrastructure inspection.

Future work will focus on conducting field trials to further validate the approach. Additional extensions could include exploring volumetric coverage using 3D partitioning strategies, e.g., by utilizing octrees [44] to capture complex underwater structures more thoroughly, as well as multi-vehicle coordination, e.g., by employing machine learning [45] to reduce mission time and increase fault tolerance.

## Figures and Tables

**Table 1:** Comparison of related CPP methods.

Decomposition Approach	Minimum Turns	Minimum Length	Line-Sweep Connection	Obstacle Avoidance Connecting Line-Sweeps	Cell Connection
Boustrophedon [14]	No	No	Back and Forth Sweeps	No	Cell Adjacency Graph
Line-Sweep-based [17]	Yes	No	Back and Forth Sweeps	No	Dynamic Programming
Iterated [19]	Yes	Yes	GTSP with Dubins’ paths	No	Redundant
Proposed	Yes	Yes	GTSP with A*	Yes	Redundant

**Table 2:** Comparison of related low-complexity PPC methodologies that consider input constraints.

Approach	Reference	Methodology	Prescribed Performance Under Saturation
Virtual Performance Constraint Control	[29]	PPC augmented with virtual performance constraints	Relaxed specifications
Adaptive PPC that modifies prescribed performance to account for saturation	[30]	PPC with adaptive performance constraints	Relaxed specifications
Adaptive Performance Control	[31]	PPC with adaptive performance constraints	Relaxed specifications
Reference Modification	[32]	PPC with modified reference trajectory	Modified tracking trajectory
Switching Control Approach	[33]	Switching controller when the input becomes saturated	Relaxed specifications
Virtual Reference Modification	[34]	PPC with modified velocity reference trajectory	Prescribed output performance

## Data Availability

Data are contained within the article.
